# Effectiveness of patient education plus motor control exercise versus patient education alone versus motor control exercise alone for rural community-dwelling adults with chronic low back pain: a randomised clinical trial

**DOI:** 10.1186/s12891-022-06108-9

**Published:** 2023-02-23

**Authors:** Aminu A. Ibrahim, Mukadas O. Akindele, Sokunbi O. Ganiyu

**Affiliations:** 1 Department of Physiotherapy, School of Basic Medical Sciences, Skyline University Nigeria, Kano State, Nigeria; 2 Department of Physiotherapy, Faculty of Allied Health Sciences, College of Health Sciences, Bayero University, Kano, P.M.B 3011, Kano State, Nigeria

**Keywords:** Chronic low back pain, Community-dwelling adults, Disability, Motor control exercise, Pain, Patient education, Rural Nigeria

## Abstract

**Background:**

Chronic low back pain (CLBP) is a common health problem in rural Nigeria but access to rehabilitation is limited. Current clinical guidelines unanimously recommend patient education (PE) including instruction on self‐management, and exercises as frontline interventions for CLBP. However, the specific content of these interventions and how they are best delivered remain to be well-described, particularly for low-resource communities. This study determined the effectiveness of PE plus motor control exercise (MCE) compared with either therapy alone among rural community-dwelling adults with CLBP.

**Methods:**

A single-blind, three-arm parallel-group, randomised clinical trial including 120 adult rural dwellers (mean [SD] age, 46.0 [14.7] years) with CLBP assigned to PE plus MCE group (*n* = 40), PE group (*n* = 40), and MCE group (*n* = 40) was conducted. The PE was administered once weekly and the MCE twice weekly. Each group also received stretching and aerobic exercises twice weekly. All interventions were administered for 8 weeks. Blinded assessments for pain intensity and disability level as primary outcomes, and quality of life, global perceived recovery, fear-avoidance beliefs, pain catastrophising, back pain consequences belief and pain medication use as secondary outcomes were conducted at baseline, 8-week (immediately after intervention) and 20-week follow-ups.

**Results:**

All the groups showed significant improvements in all the primary and secondary outcomes evaluated over time. Compared with PE alone, the PE plus MCE showed a significantly greater reduction in pain intensity by an additional –1.15 (95% confidence interval [CI], –2.04 to –0.25) points at the 8-week follow-up and –1.25 (95% CI, –2.14 to –0.35) points at the 20-week follow-up. For disability level, both PE plus MCE and MCE alone showed a significantly greater improvement compared with PE alone by an additional –5.04% (95% CI, –9.57 to –0.52) and 5.68% (95% CI, 1.15 to 10.2) points, respectively, at the 8-week follow-up, and –5.96% (95% CI, –9.84 to –2.07) and 6.57% (95% CI, 2.69 to 10.4) points, respectively, at the 20-week follow-up. For the secondary outcomes, at the 8-week follow-up, PE plus MCE showed a significantly greater reduction in fear-avoidance beliefs about physical activity compared with either therapy alone, and a significantly greater reduction in pain medication use compared with PE alone. However, compared with PE plus MCE, PE alone showed a significantly greater reduction in pain catastrophising at all follow-up time points, and a significantly greater improvement in back pain consequences belief at the 20-week follow-up. Additionally, PE alone compared with MCE alone showed a significantly greater improvement in back pain consequences belief at all follow-up time points. No significant between-group difference was found for other secondary outcomes.

**Conclusions:**

Among rural community-dwelling adults with CLBP, PE plus MCE led to greater short-term improvements in pain and disability compared with PE alone, although all intervention strategies were associated with improvements in these outcomes. This trial provides additional support for combining PE with MCE, as recommended in current clinical guidelines, to promote self-management and reduce the burden of CLBP in low-resource rural communities.

**Trial registration:**

ClinicalTrials.gov (NCT03393104), Registered on 08/01/2018.

**Supplementary Information:**

The online version contains supplementary material available at 10.1186/s12891-022-06108-9.

## Background

Low back pain remains the most common musculoskeletal and burdensome condition. It has been the leading cause of disability internationally, accounting for 63.7 million years lived with disability across all age groups since 1990 [1]. Years lived with disability due to low back pain is currently greater among individuals aged 45–54 years [2] implying working-age population is the most vulnerable group, which is more problematic in resource-constrained countries where hard physical labour and informal employment are common [3, 4]. Low back pain is also a frequent cause of activity limitation, participation restrictions, work absenteeism, and lost productivity, hence remains a major public health problem, with substantial personal and societal costs [3, 5, 6].

As the number of people with low back pain is increasing likely due to global ageing and population growth [1], there is growing concern about its impact, particularly in low and middle-income countries. This is more so like those in Sub-Saharan Africa, where most people are living in rural communities with ill-equipped health care systems to cope with the growing burden besides other priorities such as communicable diseases (e.g. malaria and HIV/AIDS) [3, 7, 8]. The 12-month low back pain prevalence rate of 33–74% estimated for Nigeria [9] is higher than the 25–70% estimated for other African nations [10] and the 20% estimated globally [11]. Moreover, the 12-month prevalence range of 40–79% [12–16] reported for rural Nigeria is disproportionally greater than the range of 38–61% reported for urban Nigeria [17–19].

Occupational biomechanical factors, such as heavy physical work, heavy manual lifting and prolonged trunk flexion mainly due to farming [12, 15, 20, 21] in addition to psychosocial factors, particularly fear-avoidance beliefs and catastrophising [4] have been associated with work-related disability due to low back pain in rural Nigeria. It was shown that more than half (52%) of farmers in rural Nigeria had reduced their farming workload and one-third of them had been absent from work in the past year due to low back pain [13]. Overall, this suggest that Nigerians, especially rural dwellers, are likely to suffer one of the greatest burdens of this disorder worldwide.

Despite the burden of low back pain in rural Nigeria, rehabilitation services are lacking even at the rural primary health care centres [22]. Consequently, the management of musculoskeletal pain in this context is predominantly a drug-based biomedical approach by patronising unconventional practitioners such as patent medicine vendors and herbalists due to their availability and affordability, and difficulty in accessing conventional health care [21, 23]. The non-availability of physiotherapy services coupled with poor referral practices by community extension workers, poor knowledge of the roles and scope of physiotherapy, poor healthcare-seeking behavior [22, 24], fear of conventional health care [21], as well as high rates of poverty [25] in rural Nigeria are obvious barriers to obtaining effective chronic low back pain (CLBP) rehabilitation.

As contemporary understanding suggests that biophysical and psychosocial factors, as well as peripheral and central processing mechanisms, play an important role in CLBP [3], it has been recommended that clinicians should embrace a biopsychosocial perspective in its management. [26] Additionally, a recent call-to-action by leading back pain researchers underscores the need to avoid low-value or harmful treatments and consider evidence-based interventions for people with CLBP taking into account costs, availability of interventions, and cultural and patient preferences [8, 27]. Non-pharmacological therapies are the mainstay of CLBP treatment, and current clinical guidelines unanimously recommend providing patient education (PE) including instruction on self-management, and exercises as frontline interventions [28, 29]. However, the specific content of these interventions and how they are best delivered remain to be well-described, particularly for low-resource communities.

Patient education, a common intervention strategy for the prevention and management of low back pain, is typically used to modify the beliefs and behaviors of patients to improve their health outcomes [30]. It is a key intervention component to empower patients with the appropriate skills to take more control over their health condition [31], which is highly relevant for patients with long-term conditions such as CLBP to minimise health care utilisation. Although different forms of PE for low back pain exist, most strategies conventionally fall into biomedical education (i.e. back school) and biopsychosocial education [32, 33]. However, previous trials [34, 35] and reviews [36, 37] suggest that biopsychosocial PE approach (i.e. education programme incorporating at least cognitive-behavioral self-management strategies), which is in line with contemporary understanding of pain [38], maybe more effective compared to purely biomedical-based PE for CLBP.

Compelling evidence suggests that exercise therapy is moderately effective for CLBP [39, 40], yet there is still uncertainty about the most optimal approach [41]. Therefore, it appears that the choice of exercise probably depends on the therapist’s preferences, skills, costs and safety [42]. Nonetheless, one promising exercise training that has been a major focus in low back pain rehabilitation is motor control exercise (MCE) [42]. This specific exercise training was developed based on the evidence that individuals with low back pain tend to exhibit delayed onset of activity of the deep trunk muscles (e.g. transversus abdominis) in dynamic tasks that challenge the control of the spine [43–45], atrophy and a large percentage of fat infiltrations in the lumbar multifidus [46–48], as well as a strategy for increased stiffness and stability at the expense of spinal function [49]. MCE is therefore, applied using principles of motor learning such as segmentation, simplification and activity to correct these deficiencies by rehabilitating the posture, movement and coordination of the trunk muscles [50]. This may in turn help to alleviate pain and symptoms associated with low back pain [42]. The advantages of MCE are that it is easy to learn though it may be quite challenging and does not require special equipment, unlike resistance exercises, hence, patients can independently practice at home which is crucial for self-management. Importantly, several systematic reviews/meta-analyses supported its efficacy in alleviating pain and disability [51, 52].

In light of the growing burden of CLBP and the non-availability of rehabilitation services in rural Nigeria, there is a pressing need to determine safe, effective and affordable guideline-endorsed interventions to help rural dwellers suffering from this disabling condition. Delivering low-cost evidence-based interventions to communities with limited or no access to health care would help to prevent harmful treatments. Our previous pilot study [53] indicated the feasibility of a full-scale, physiotherapist-led randomised clinical trial (RCT) to test the effectiveness of a combined structured group-based PE (specifically focusing on postural hygiene, pain education, coping strategies, psychological and behavioural lifestyle factors, and self-care skills) with supervised MCE aiming at teaching rural dwellers with CLBP how to self-manage their pain and functional incapacity. Notably, the results of the study showed that patients receiving PE plus MCE had their pain and disability reduced by 67.6% and 46.6% respectively compared with those receiving PE alone (38.3% and 31.7% for pain and disability respectively), or MCE alone (50.0% and 26.9% for pain and disability respectively) [53]. These preliminary findings, however, need to be confirmed in a well-designed RCT.

The primary objective of this study was to determine the effectiveness of PE plus MCE compared with either therapy alone on pain intensity and disability level among rural community-dwelling adults with CLBP. Secondary objectives were to compare the effectiveness of these interventions on quality of life (QoL), perceived recovery, fear-avoidance beliefs, pain catastrophising, back pain consequences belief and pain medication use. We hypothesised that participants receiving PE plus MCE would demonstrate greater improvements in pain intensity and disability level compared with those receiving either therapy alone.

## Materials and methods

### Study design

This study was a single-blind, three-arm, parallel-group RCT conducted between March 2018 and January 2020. It was registered prospectively at ClinicalTrials.gov (NCT03393104) on 08/01/2018 and reported according to the recommendations of the Consolidated Standards of Reporting Trials (CONSORT) guidelines [54]. A detailed study protocol has been previously published [55]. No major changes different from the published protocol were made in the present study.

### Study setting

This study was conducted at Tsakuwa Primary Health Care Centre in Tsakuwa town, Dawakin-Kudu Local Government Area, Kano State, Northwestern Nigeria.

### Study population

Multiple village-wide announcements facilitated by village/ward heads (traditional rulers) and adverts via local posters pasted at the research centre and different locations in the community were used to recruit participants until the target sample size was achieved. Potential participants were invited to the primary health care centre and upon their arrival, eligibility was ensured using the stipulated study inclusion and exclusion criteria. The eligibility assessments were carried out by physiotherapists with the use of body charts to identify pain in the lower back, history taking, and screening to rule out ‘red flags’ for low back pain [56]. All participants provided written informed consent. Inclusion criteria were as follows: (1) male or female between the age of 18 and 70 years, (2) nonspecific low back pain with or without leg pain experienced for 12 weeks or more, and (3) ability to read and understand Hausa or English language or both. Exclusion criteria were: (1) history of spine surgery, (2) obvious spine or limb deformities, (3) serious spinal pathology (e.g. infection, metastases, cauda equina syndrome, and fracture) (4) unstable or severe disabling chronic cardiovascular and pulmonary diseases, (5) inadequate visual and hearing ability, (6) previous physiotherapy treatment in the form of exercise and/or education in the last 3 months, (7) body mass index (BMI) ≥ 35 kg/cm^2^, and (8) pregnancy. Verbal information about the study purpose and potential benefits using an information sheet was read to the eligible participants. Those willing to participate were then given a written informed consent form to sign or make a thumbprint.

### Sample size estimation

Minimal clinically important difference (MCID) of 2.0–4.0 points for pain measured by numerical pain rating scale (NPRS) and 5.0–17.0% points for disability measured by Oswestry disability index (ODI) have been reported for low back pain studies [57, 58]. Informed by our pilot study [53], this study was powered to detect a minimum difference of 1.0 (standard deviation [SD] = 1.3) points in pain or 5.0% (SD = 6.3%) points in disability between highest and lowest group means after intervention. With a moderate effect size of 0.32, an alpha level of 0.05, a statistical power of 90%, a correlation of 0.5 among repeated measures, and a potential dropout rate of 40%, it was necessary to recruit 120 participants (*n* = 40 per group). Calculations were performed with the G-power 3.1.9.2 software (University of Dusseldorf, Dusseldorf, Germany) [59].

### Randomisation and blinding

After completing all baseline assessments, the consenting participants were randomly assigned to one of three groups; PE plus MCE, PE alone or MCE alone in a 1:1:1 ratio using a block randomisation procedure. A web-based randomisation tool (www.sealedenvelope.com) was used to generate a randomisation sequence with randomly permuted blocks of varying sizes (3, 6 and 9) by a third party. The random allocation sequence list was handed over to a record officer in the study centre, who was not involved in any other aspects of the study, for the random assignment. The executor kept the randomisation sequence secret. Upon certifying the eligibility of participants, randomisation was requested. Consecutive consented participants were assigned to the three study groups according to the predetermined matched block. The allocation of participants was concealed from the research assistants enrolling and assessing participants by not revealing the block sizes and by using sequentially sealed and stapled envelopes. All outcome assessors were kept blinded to the size of each block and randomisation list. The participants were instructed not to reveal their groups to the outcome assessors. However, it was difficult to blind the treating therapists to treatment allocation due to the nature of the study interventions. 

### Study interventions

The interventions commenced immediately after baseline assessments and randomisation. Participants in the PE plus MCE group received PE followed by stretching, MCE and aerobic exercise. Participants in the PE alone group received PE followed by stretching and aerobic exercise, whereas those in the MCE received stretching followed by MCE and aerobic exercise. The PE was administered once a week for 8 weeks (8 sessions), whereas stretching and MCE were administered twice a week for 8 weeks (16 sessions). Participants were treated on alternate days to minimise cross-contamination between groups. All exercises except aerobics (non-supervised continuous overground walk or bicycling) were supervised by physiotherapists. All the participants were advised to refrain from receiving other interventions such as herbal preparations and treatment from traditional bonesetters to prevent contamination of outcome. However, prescribed pain tablets were permissible as it would be unethical to withhold medication. They were encouraged to perform exercises consistent with their group treatment at home. To enhance self-management, participants were given a booklet (translated into Hausa) according to the group they were assigned. Participants in the PE plus MCE received a booklet containing key information about the PE programme (including picture models of postural hygiene and relaxation positions) and picture models of stretching and MCE resembling rural northern Nigerian dwellers. Participants in the PE alone group received a booklet consisting of the aforementioned components but without the MCE programme. Those in the MCE alone group however received a booklet containing only the stretching and MCE programme. All participants were encouraged to continue with home exercises at the end of the study to sustain self-management. The researcher kept in touch with the participants through phone calls to remind them of their follow-ups from time to time.

### Patient education

A face-to-face, group biopsychosocial-based PE session was delivered for 8 weeks by the lead author in the local language (Hausa). The content of the education programme was guided by available evidence on advice and education for patients with CLBP [36, 37, 60–67] and developed in close collaboration with experienced physiotherapists. Based on the outcome of our pilot study [53], the PE programme had a couple of modifications before it was finally delivered. The PE was developed to support the MCE programme and build motivation and ability to sustain the MCE after the 8-week study. As fully described in the protocol paper [55], educational sessions covering different topics were delivered once weekly for 8 consecutive weeks. During each session, motivation and reinforcement were provided. At the end of each session, participants were allowed to ask questions, and areas requiring additional explanation were reviewed. Overall understanding or success with the PE programme was assessed during the refresher session (8th week). Topics requiring further explanation were readdressed. An overview of the PE programme is provided in Table 1. The PE programme typically lasted between 60 and 80 min per session.

**Table 1:** Overview of the patient education programme

Session/week	Topic	Goals/objectives
1	a. Interactive session/discussions/questions b. Meaning of low back pain c. Common myths and facts about low back pain d. Common beliefs about low back pain	a. To establish good rapport and explore participants' beliefs about low back pain b. To promote a better understanding of low back pain c. To understand the common myths and facts about low back pain d. To reshape false or unhelpful beliefs about low back pain
2	a. Basic anatomy b. Pain causation	a. To promote understanding of the robustness and function of the spine b. To promote a better understanding of the cause of pain
3	a. Basic pain education (part 1) b. Basic pain education (part 2	a. To promote basic knowledge about pain mechanisms from modern pain models b. To educate on the common factors associated with pain experience
4	a. Resumption of work and remaining active b. Pain coping and pacing	a. To encourage the resumption of normal activities and the importance of remaining active despite pain b. To promote better active coping through adopting safe and effective pacing during flare-ups
5	Self-care skills	To promote self-management strategies and reduce overreliance on formal health care utilisation
6	a. Postural modification b. Increasing activity levels	a. To promote healthy postural habits using current evidence on ergonomics b. To promote the importance of improving activity levels
7	a. Lifestyle modification b. Warning signs and what to do	a. To promote a healthy lifestyle b. Promote understanding of red flags and the importance of hospital visit
8	Review of information and application	To evaluate understanding and application of information

### Stretching exercises

The stretching exercises used in this study were fully described in our pilot study [53]. The participants received 9 different stretching exercises aiming to increase lumbopelvic-hip flexibility (Table 2) (see Supplementary Fig. 1, Additional file 1). The stretching exercises lasted for 20 min per session.

**Table 2:** Overview of the exercise interventions of the study

Exercise	PE Plus MCE group	PE group	MCE group	Intensity	Sessions	Week
**Motor control exercise**						
ADIM in supine	✓		✓	7 s hold, 10 reps	1st–4th (Stage I)	1–2
ADIM in quadruped	✓		✓	✓		
ADIM in sitting	✓		✓	✓		
ADIM in standing	✓		✓	✓		
ADIM in supine with leg lift (each leg)	✓		✓	7 s hold, 10 reps	5th–14th (Stage II)	3–6
ADIM in supine with bridging (two legs)	✓		✓	✓		
ADIM in supine with single-leg bridge	✓		✓	✓		
ADIM in supine with curl-up (elbows on the table)	✓		✓	✓		
ADIM in supine with curl-up (hands over the forehead)	✓		✓	✓		
ADIM in horizontal side support with knees bent	✓		✓	✓		
ADIM in horizontal side support with knees straight	✓		✓	✓		
ADIM in side-lying horizontal side support	✓		✓	✓		
ADIM in quadruped with arm raise	✓		✓	✓		
ADIM in quadruped with leg raise	✓		✓	✓		
ADIM in quadruped with alternate arm and leg raise	✓		✓	✓		
Rolling from side to side with ADIM	✓		✓	✓	15th and 16th (Stage III)	7 and 8
Sit-stand transfer with ADIM	✓		✓	30 reps		
Wall squatting with ADIM	✓		✓	✓		
Progressive walking with ADIM (10 min)	✓		✓	7 s hold, 10 s relax, 10 reps		
**Stretching exercise**						
Double knee to chest stretch	✓	✓	✓		1st–16th	1–8
Piriformis stretch	✓	✓	✓		✓	✓
Hamstring stretch	✓	✓	✓		✓	✓
Trunk rotation	✓	✓	✓		✓	✓
Cat pose stretch	✓	✓	✓		✓	✓
Prone on elbow	✓	✓	✓		✓	✓
Hip adductor stretch	✓	✓	✓		✓	✓
Triceps surae stretch	✓	✓	✓		✓	✓
Trunk extension stretch	✓	✓	✓		✓	✓
**Aerobic exercise**						
Overground walk or bicycling etc	✓	✓	✓		NA	1–8

*ADIM* Abdominal drawing-in manoeuvre, *NA* Not available

### Motor control exercises

The MCE programme was designed to improve function of specific muscles of the lumbopelvic region and control of posture and movement [50, 68]. The content of the programme was based on previous studies [68, 69] demonstrating promising benefits of MCE with a detailed description of the training programme reported in the study protocol [55] after being tested in our pilot study [53]. Exercises were performed in three stages over 8 weeks (Table 2) (see Supplementary Fig. 2, Additional file 2). Although exercise sessions were conducted in groups, the progression of exercises was based on the patient's fatigue, pain thresholds, or observed movement control. The MCE programme typically lasted for 30 min per session.

### Aerobic exercise

Participants were advised to perform a preferred aerobic exercise such as overground walk or bicycling at a desirable speed at home for a minimum of 30 min, 5 times per week (Table 2). The purpose was to encourage aerobic activity and improve cardiorespiratory fitness as per the recommendation of the American College of Sports Medicine [70].

### Outcome measures

Participants’ demographic data were collected using researcher-designed data forms in line with the recommendation of the National Institute of Health (NIH) task force on research standards for CLBP. Primary outcomes (pain intensity and disability level) and secondary outcomes (QoL, global perceived recovery, fear-avoidance beliefs, pain catastrophising, back pain consequences belief and pain medication use) were assessed at baseline, 8-week (immediately after intervention) and 20-week follow-ups by trained physiotherapists, blinded to group allocation. All outcomes except pain medication use were obtained using self-report questionnaires as described in detail in the protocol paper [55].

### Primary outcomes

Pain intensity was assessed using the reliable, valid and responsive Hausa version of the numerical pain rating scale (NPRS) [71]. It is scored 0–10 cm, where 0 indicates no pain and 10 indicates worst imaginable pain. Disability due to low back pain was assessed using the reliable and valid Hausa version of the Oswestry disability index (ODI) 2.1a [72]. It is scored 0–50 and recorded as percentage (0–100), with higher scores indicating higher disability [72].

### Secondary outcomes

Quality of life was assessed using the reliable and valid Hausa version of the 12-item short-form health survey (SF-12) [73]. Physical component summary (PCS) and mental component summary (MCS) subscales were used to determine physical and mental health respectively, with higher scores indicating better health status [73]. Global perceived recovery was assessed using the reliable and valid Hausa version of the 11-point global rating of change scale **(**GRCS) [71]. It is rated from –5 to + 5 with a mid-point (0) representing “no change”, a left anchor (–5) representing “Very much Worse”, and a right anchor (+ 5) representing “Completely Recovered” [71]. Fear-avoidance belief were assessed using the reliable and valid Hausa version of the fear-avoidance beliefs questionnaire **(**FABQ) [74]. It has two subscales, FABQ-physical activity (scored 0–24) and FABQ-work (scored 0–42), with higher scores indicating greater fear-avoidance beliefs [74]. Pain catastrophising was assessed using the reliable and valid Hausa version of the pain catastrophising scale (PCS) [75]. It is scored 0–52, with higher scores indicating more catastrophic thoughts. [75]. Back pain consequences belief were assessed using the reliable and valid Hausa version of the back beliefs questionnaire (BBQ) [76]. It is scored 9–45, with nine items (1, 2, 3, 6, 8, 10, 12, 13, and 14) after reversing. Higher scores indicate less pessimistic beliefs regarding the consequences of back pain [76]. Pain medication use was assessed by recording the number of pain tablets ingested in the past 4 weeks (prior to commencement of the study) at baseline and 8-week follow-up. The participants were asked to record their pain medication use with the use of a diary, or present their medication sachet pack if available in case they could not use a diary.

### Adverse events

Participants in all the groups were asked to document any serious adverse events related to the study interventions during the intervention and follow-up periods and report to the project coordinator.

### Statistical analyses

All statistical analyses were performed on IBM SPSS version 24.0 (IBM Co., Armonk, NY, USA) at an alpha level of 0.05. Data were summarised using mean (SD) for continuous variables and frequency (percentage) for discrete variables. Comparison of baseline categorical variables among the different treatment groups (PE plus MCE, PE, MCE) was conducted using the chi-square test (for cells count > 5) or Fisher's exact test (for cells count < 5) while one-way between-subjects analysis of variance (ANOVA) was used for continuous variables.

### Intention to treat analysis

Intention-to-treat analysis was the main analysis and performed with randomised participants included in the treatment groups in which they were originally allocated [77]. Missing data (< 5% of the overall responses) were handled using multiple imputation method [78]. Intervention effects on primary and secondary outcome measures were analysed using linear mixed-effects models (LMMs) fitted by maximum likelihood approach for parameter estimation. Separate models for each primary and secondary outcome were computed with time (baseline, 8 weeks, 20 weeks), treatment group (PE plus MCE, PE, MCE) and a treatment group by time interaction as fixed effects, and subjects (participants) as random effect to model the within-subject correlations. Treatment effect was summarised as the adjusted between-group difference and the associated 95% confidence intervals (CI) (from estimated marginal means command) at 8 and 20 weeks with respect to the baseline. Bonferonni adjustment with an alpha of 0.017 (i.e. α = 0.05/3) was applied for pairwise comparisons for the primary outcomes (pain intensity and disability level), whereas Fisher's least significant difference (equivalent to no adjustments) with the conventional alpha of 0.05 was applied for pairwise comparisons for the secondary outcomes.

### Sensitivity analysis

As part of the sensitivity analysis, additional intention-to-treat LMMs analyses for the primary outcomes were conducted while adjusting for age, gender, BMI, low back pain duration, and educational level. Responder analyses for the number of participants reporting ≥ 30% (MCID) reduction from baseline in pain intensity and disability level [57, 58] were conducted at 8- and 20-week follow-ups. Participants in each group were dichotomised into two “benefit” (≥ 30%) and “no benefit” (< 30%). A chi-square test was conducted to compare the proportion of each group’s participants reaching or not reaching MCID after intervention. Further, per-protocol analysis was performed by excluding randomised participants who did not attend any session, or attended fewer than 10 MCE sessions and 5 PE sessions, received prohibited concomitant intervention for low back pain, and developed exclusionary medical conditions as listed in the study exclusion criteria. LMMs were also applied for the per-protocol analysis.

## Results

### Sociodemographic characteristics of the participants

**Fig. 1:**
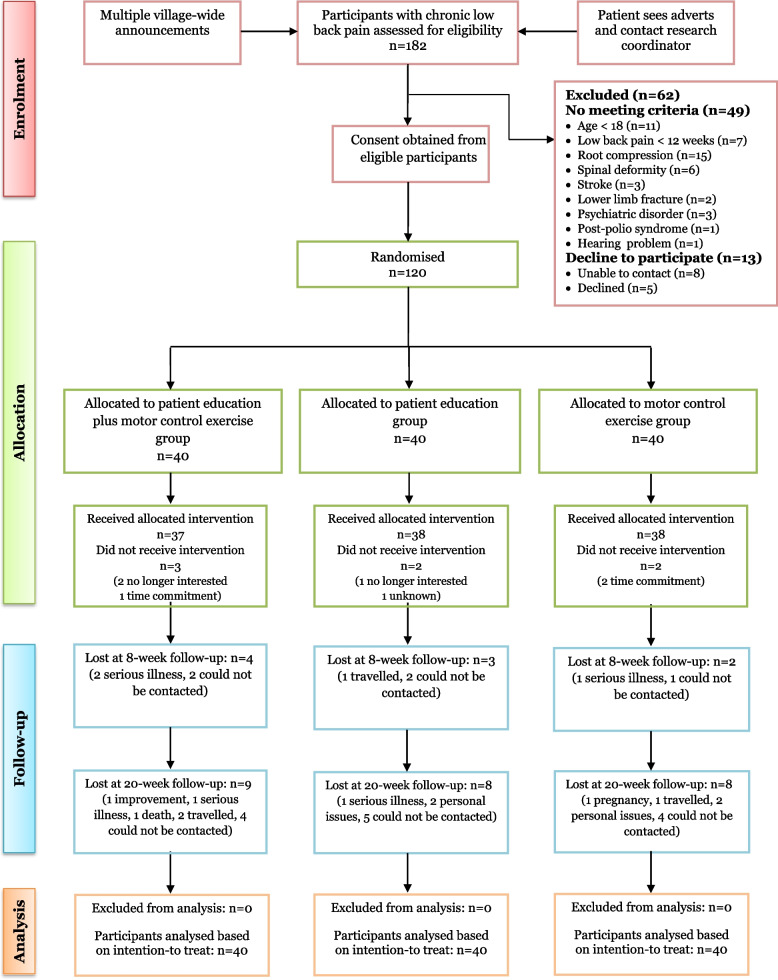
Flow of participants through the study

A summary of the participants’ enrolment, treatment adherence, and attrition during the study is depicted in Fig. 1. From March 2018 through January 2020, 182 individuals with CLBP were screened, 62 were ineligible, and 120 participants (72 men [60%] and 48 women [40%]) with a mean (SD) age of 46.0 (14.7) years met all the inclusion criteria and were recruited. The majority of them were non-literate in formal education (66.7%) and self-employed (81.7%). Only two of the participants (1.7%) had previous experience with physiotherapy but over the past 3 months. The three groups were comparable in all the baseline variables (*P* > 0.05). Sociodemographic and baseline clinical characteristics of all randomised participants are fully shown in Table 3.

**Table 3:** Participants’ baseline sociodemographic and clinical variables

Variables	PE plus MCE (*n* = 40)	PE (*n* = 40)	MCE (*n* = 40)	*P value*	Total (*n* = 120)
Age (y), mean (SD)	45.0 (15.2)	47.78 (15.9)	45.1 (13.1)	0.653^a^	46.0 (14.7)
BMI (kg/m^2^), mean (SD)	21.7 (2.81)	22.30 (3.88)	23.3 (4.44)	0.164^a^	22.4 (3.78)
Marital status, *n* (%)					
Married	35 (87.5)	32 (80.0)	36 (90)	0.671^c^	103 (85.8)
Single	4 (10.0)	5 (12.5)	3 (7.5)		12 (10.0)
Widow	1 (2.5)	3 (7.5)	1 (2.5)		5 (4.2)
Gender, *n* (%)					
Male	26 (65.0)	26 (65.0)	20 (50.0)	0.287^b^	72 (60.0)
Female	14 (35.0)	14 (35.0)	20 (50.0)		48 (40.0)
Educational status, *n* (%)					
Non-formal education	23 (57.5)	32 (80.0)	25 (62.5)	0.094^c^	80 (66.7)
Completed primary education	12 (30)	3 (7.5)	6 (15.0)		21 (17.5)
Completed secondary education	4 (10.0)	2 (5.0)	6 (15.0)		12 (10.0)
Completed tertiary education	1 (2.5)	3 (7.5)	3 (7.5)		7 (5.8)
Occupational status, *n* (%)					
Paid work (government or private)	2 (5.0)	5 (12.5)	2 (5.0)	0.495^c^	9 (7.5)
Self-employed (farming or trading)	35 (87.5)	30 (75.0)	33 (82.5)		98 (81.7)
Student	1 (2.5)	2 (5.0)	4 (10.0)		7 (5.8)
Unemployed	2 (5.0)	3 (7.5)	1 (2.5)		6 (5.0)
Pain duration, *n* (%)					
3 – 24 months	16 (40.0)	12 (30.0)	16 (40.0)	0.563^b^	43 (35.8)
> 24 months	24 (60.0)	28 (70.0)	24 (60.0)		77 (64.2)
Smoking status, *n* (%)					
Yes	1 (2.5)	1 (2.5)	2 (5.0)	0.772^c^	116 (96.7)
No	39 (97.5)	39 (97.5)	38 (95.0)		4 (3.3)
Hypertension, *n* (%)				0.646^c^	
Yes	5 (11.6)	6 (15.0)	8 (20.0)		19 (15.8)
No	35 (81.4)	34 (85.0)	32 (80.0)		101 (84.2)
Systolic BP (mmHg), mean (SD)	127.6 (19.7)	129.2 (19.7)	128.1 (17.4)	0.926^a^	128.3 (18.8)
Diastolic BP (mmHg), mean (SD)	86.6 (8.50)	85.5 (9.11)	85.3 (7.62)	0.767^a^	85.8 (8.38)
Diabetes, *n* (%)				0.466^c^	
Yes	2 (2.70)	3 (7.5)	5 (12.5)		10 (8.30)
No	38 (88.4)	37 (92.5)	35 (87.5)		110 (91.7)
Previous physiotherapy (**> **3 months), *n* (%)					
Yes	1 (2.5)	1 (2.5)	0 (0.0)	0.601^c^	2 (1.7)
No	39 (97.5)	39 (97.5)	40 (100.0)		118 (98.3)

*PE* Patient education, *MCE* Motor control exercise, *SD* Standard deviation, *BMI* Body mass index, *BP* Blood pressure

^a^Analysed by one-way analysis of variance

^b^Analysed by chi-square test

^c^Analysed by Fisher’s exact test

### Treatment adherence and adverse events

Adherence to intervention was high with 92.5% in the PE plus MCE group (37/40) and 95% in both the PE alone group (38/40) and MCE alone group (38/40) receiving the allocated interventions (Fig. 1). The mean (SD) number of PE sessions attended was 4.7 (1.6) in the PE plus MCE group and 4.8 (1.9) in the PE alone group. The mean (SD) number of MCE sessions was 9.1 (2.7) in the PE plus MCE group and 9.2 (2.8) in the MCE alone group. During the 8-week intervention period, 9 participants (PE plus MCE group = 4, 10.0%; PE alone group = 3, 7.5%; and MCE alone group = 2, 5.0%) withdrew from the study. Additionally, 25 participants (PE plus MCE group = 9, 27.2%; PE alone group = 8, 22.2%; and MCE alone group = 8, 22.8%) withdrew during the 20-week follow-up (Fig. 1). Most of the participants lost to follow-up during the trial could not be contacted. Six participants had serious adverse events, but were all evaluated to be unrelated to the study interventions. In the PE plus MCE group, one had motorcycle accident, one died from stroke, and two were hospitalised (one for herniotomy and the other for appendicectomy). In the PE alone group, one was newly diagnosed with prostate cancer. In the MCE alone group, one had exacerbating pain due to hip fracture caused by a fall.

### Intervention effectiveness

#### Intention to treat analysis for primary outcomes

**Fig. 2:**
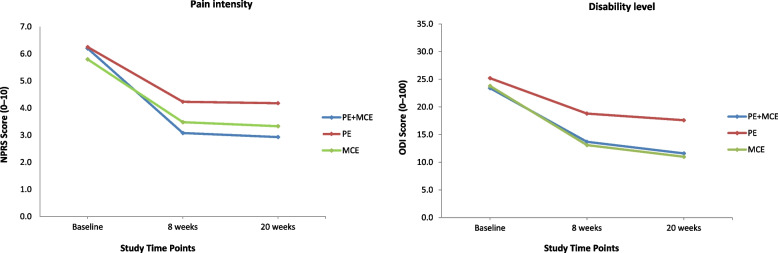
Pain intensity scores and disability level for all groups across time. Notes: PE indicates patient education, and MCE indicates motor control exercise

The mean pain intensity (scale of 0 to 10) at baseline was similar among the three groups. Pain intensity significantly decreased in all the three groups by 2.0–3.1 points at the 8-week follow-up and by 2.1–3.3 points at the 20-week follow-up (Fig. 2 and Table 4). However, compared with the PE alone group, the PE plus MCE group showed a significant treatment effect with reduced pain intensity by an additional –1.15 (95% CI, –2.04 to –0.25) points at the 8-week follow-up and –1.25 (95% CI, –2.14 to –0.35) points at the 20-week follow-up (Table 4). Similarly, the mean disability level (scale of 0 to 100) at baseline was comparable among the three groups and significantly reduced by 6.4–10.7% points at the 8-week follow-up and by 7.6–12.8% points at the 20-week follow-up (Fig. 2 and Table 4). However, compared with the PE alone group, both PE plus MCE and MCE alone groups showed a significant treatment effect with reduced disability by an additional –5.04% (95% CI, –9.57 to –0.52) and 5.68% (95% CI, 1.15 to 10.2) points respectively, at the 8-week follow-up, and –5.96% (95% CI, –9.84 to –2.07) and 6.57% (95% CI, 2.69 to 10.4) points respectively, at the 20-week follow-up (Table 4).

**Table 4:** Primary and secondary outcomes measured at baseline, 8 and 20 weeks with between-group comparisons based on intention-to-treat principle

**Outcome**	**Treatment group** Mean (SD)			**Adjusted between-group difference** Mean (95% CI)		
	PE + MCE	PE	MCE	PE + MCE vs PE	PE + MCE vs MCE	PE vs MCE
**Primary outcomes**						
**NPRS (0–10)**						
Baseline	6.20 (1.71)	6.25 (1.83)	5.80 (1.63)	–	–	–
8 weeks	3.08 (1.65)	4.23 (1.70)	3.48 (1.73)	–1.15 (–2.04 to –0.25)^b^	–0.40 (–1.29 to 0.49)	0.75 (0.14 to 1.64)
20 weeks	2.93 (1.62)	4.18 (1.73)	3.33 (1.54)	–1.25 (–2.14 to –0.35)^b^	–0.40 (–1.29 to 0.49)	0.85 (–0.04 to 1.74)
**ODI (0–100)**						
Baseline	23.4 (12.5)	25.2 (14.5)	23.8 (12.1)	–	–	–
8 weeks	13.7 (6.77)	18.8 (10.7)	13.1 (7.21)	–5.04 (–9.57 to –0.52)^a^	0.63 (–3.88 to 5.16)	5.68 (1.15 to 10.2)^b^
20 weeks	11.6 (5.12)	17.6 (9.99)	11.0 (5.58)	–5.96 (–9.84 to –2.07)^b^	0.61 (–3.26 to 4.49)	6.57 (2.69 to 10.4)^b^
**Secondary outcomes**						
**PCS-12 (0–100)**						
Baseline	35.1 (8.36)	35.2 (8.05)	35.9 (7.97)	–	–	–
8 weeks	43.3 (7.84)	41.7 (7.99)	43.4 (7.43)	1.60 (–1.66 to 4.86)	–0.16 (–3.41 to 3.10)	–1.76 (–5.02 to 1.50)
20 weeks	45.5 (6.85)	44.5 (6.82)	45.6 (5.92)	1.03 (–2.23 to 4.29)	–0.13 (–3.40 to 3.13)	–1.16 (–4.43 to 2.10)
**MCS-12 (0–100)**						
Baseline	41.4 (8.19)	40.9 (10.9)	43.0 (9.01)	–	–	–
8 weeks	46.2 (6.59)	47.8 (9.20)	47.6 (6.11)	–1.55 (–5.01 to 1.90)	–1.43 (–4.89 to 2.01)	0.11 (–3.34 to 3.57)
20 weeks	46.6 (6.21)	45.2 (7.81)	46.6 (6.08)	1.20 (–4.66 to 2.25)	–0.14 (–3.60 to 3.30)	–1.35 (–4.80 to 2.10)
**GRCS (–5 to + 5)**						
Baseline	–1.25 (1.94)	–1.35 (1.81)	–1.25 (1.82)	–	–	–
8 weeks	1.92 (2.12)	1.80 (1.97)	2.05 (1.99)	0.12 (–0.71 to 0.96)	–0.12 (0.96 to 0.71)	–0.25 (–1.09 to 0.59)
20 weeks	2.20 (2.22)	1.35 (1.71)	1.82 (1.72)	0.85 (0.00 to 1.69)	0.37 (–0.46 to 1.21)	–0.47 (–1.31 to 0.36)
**FABQ-PA (0–24)**						
Baseline	11.6 (7.24)	13.7 (6.93)	13.9 (6.53)	–	–	–
8 weeks	6.78 (5.44)	9.98 (5.46)	9.35 (5.25)	–3.20 (–5.71 to –0.68)^a^	–2.57 (–5.09 to –0.05)^a^	0.62 (–1.89 to 3.14)
20 weeks	6.28 (4.76)	7.77 (4.87)	8.40 (4.95)	–1.49 (–4.01 to 1.01)	–2.12 (–4.64 to 0.39)	–0.62 (–3.14 to 1.89)
**FABQ-W (0–42)**						
Baseline	24.5 (8.15)	24.3 (8.45)	21.6 (10.5)	–	–	–
8 weeks	17.2 (7.70)	15.1 (8.65)	15.2 (7.37)	2.10 (–1.39 to 5.59)	2.02 (–1.46 to 5.51)	–0.07 (–3.56 to 3.41)
20 weeks	15.0 (7.46)	12.5 (7.17)	11.7 (5.97)	3.17 (–0.31 to 6.66)	3.57 (0.08 to 7.06)	0.39 (–3.09 to 3.89)
**PCS (0–52)**						
Baseline	31.3 (8.03)	29.2 (7.58)	31.0 (8.69)	–	–	–
8 weeks	22.1 (7.74)	17.2 (7.02)	19.8 (6.97)	4.85 (1.69 to 8.01)^b^	2.25 (–0.91 to 5.41)	–2.60 (–5.76 to 0.56)
20 weeks	20.4 (7.23)	15.3 (6.57)	18.3 (4.95)	5.15 (1.99 to 8.31)^b^	2.16 (–0.99 to 5.32)	–2.98 (–6.14 to 0.17)
**BBQ (9–45)**						
Baseline	17.5 (5.07)	17.9 (5.50)	18.4 (5.63)	–	–	–
8 weeks	25.6 (6.68)	28.2 (7.44)	24.4 (6.28)	–2.60 (–5.37 to 0.15)	–1.21 (–1.54 to 3.98)	3.82 (1.06 to 6.58)^b^
20 weeks	26.4 (6.90)	30.1 (7.25)	25.6 (6.01)	–3.74 (–6.50 to –0.98)^b^	0.78 (–1.97 to 3.54)	4.52 (1.76 to 7.29)^b^
**Pain medication use**						
Baseline	10.0 (8.14)	9.10 (6.81)	11.2 (8.51)	–	–	–
8 weeks	5.05 (6.77)	9.37 (7.68)	7.10 (7.00)	–4.32 (–7.59 to –1.05)^a^	–2.05 (–5.32 to 1.22)	2.27 (–0.99 to 5.54)

*CI* Confidence interval, *PE* Patient education, *MCE* Motor control exercise, *NPRS* Numerical pain rating scale, *ODI* Oswestry disability index, *PCS-12* Physical component summary-12, *MCS-12* Mental health component summary-12, *GRCS* Global rating of change scale, *FABQ-PA* Fear-avoidance beliefs questionnaire – (physical activity), *FABQ-W* Fear-avoidance beliefs questionnaire – (work), *PCS* Pain catastrophising scale, *BBQ* Back beliefs questionnaire, *NA* Not applicable

Lower scores in NPRS, ODI, FABQ-PA, FABQ-W, and PCS indicate better improvement

Higher scores in PCS-12, MCS-12, GRCS, and BBQ indicate better improvement

Participants in PE plus MCE group = 40, PE group = 40, and MCE group = 40

^a^comparison is significant at the 0.05 level,

^b^comparison is significant at the 0.01 level

#### Intention to treat analysis for secondary outcomes

**Fig. 3:**
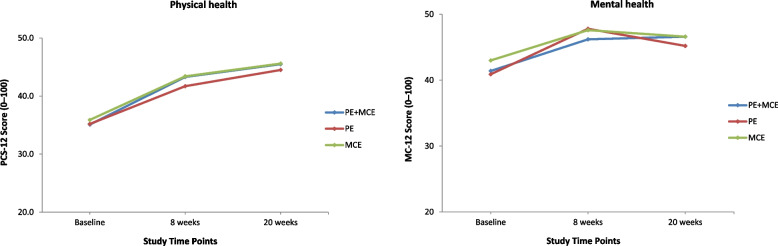
Physical health and mental health scores for all groups across time. Notes: PE indicates patient education, and MCE indicates motor control exercise

**Fig. 4:**
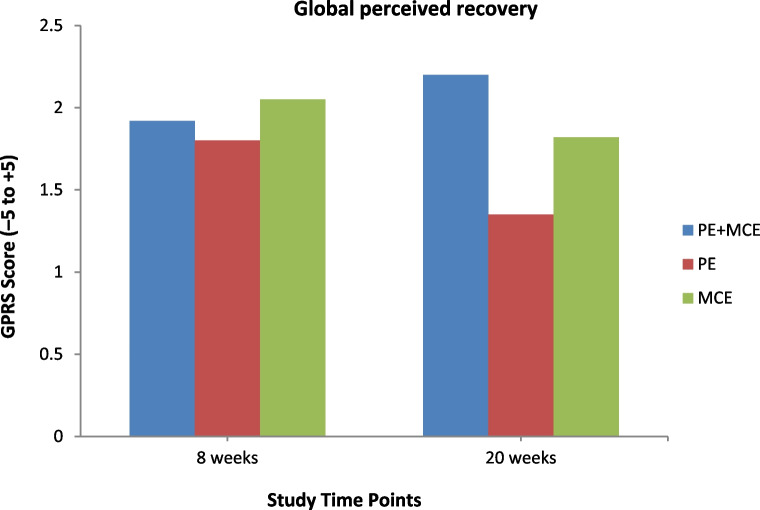
Global perceived recovery scores for all groups across time. Notes: PE indicates patient education, and MCE indicates motor control exercise

**Fig. 5:**
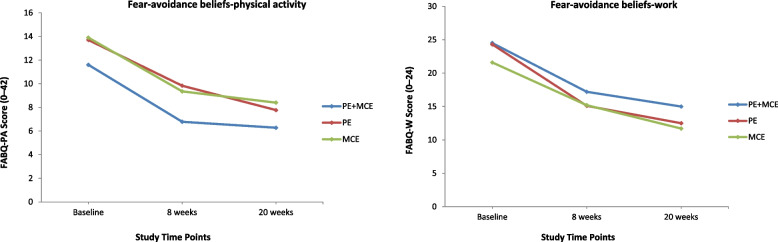
Fear-avoidance beliefs about physical activity and work scores for all groups across time. Notes: PE indicates patient education, and MCE indicates motor control exercise

**Fig. 6:**
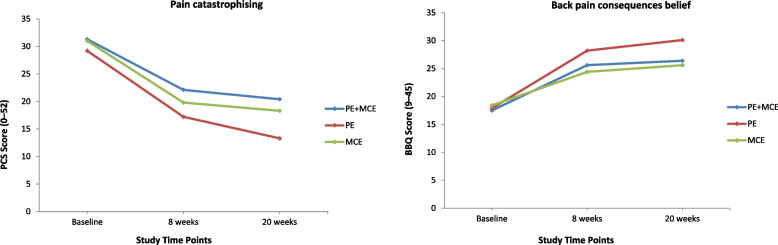
Pain catastrophising and back pain consequences belief scores for all groups across time. Notes: PE indicates patient education, and MCE indicates motor control exercise

All the secondary outcomes at baseline were similar among the three groups and showed significant improvements at all follow-up time points except that MCS-12 scores for the PE alone and MCE alone groups levelled off at the 20-week follow-up (Figs. 3, 4, 5 and 6). At the 8-week follow-up, the PE plus MCE group showed a significant treatment effect with reduced FABQ-PA scores by an additional –3.20 (95% CI, –5.71 to –0.68) points compared with the PE alone group and an additional –2.57 (95% CI, –5.09 to –0.05) points compared with the MCE alone group. Additionally, the PE plus MCE group showed a significant treatment effect with reduced pain medication use by an additional –4.32, 95% CI: –7.59 to –1.05) points compared with the PE alone group (Table 4). However, compared with the PE plus MCE group, the PE alone group showed a significant treatment effect with reduced PCS scores by an additional 4.85 (95% CI, 1.69 to 8.01) points at the end of 8-week follow-up and 5.15 (95% CI, 1.99 to 8.31) points at the 20-week follow-up, and improved BBQ scores by an additional –3.74 (95% CI, –6.50 to –0.98) points at the 20-week follow-up. The PE alone group compared with the MCE alone group again showed significantly improved BBQ scores by an additional 3.82 (95% CI, 1.06 to 6.58) points at the end of 8-week follow-up and 4.52 (95% CI, 1.76 to 7.29) points at the 20-week follow-up (Table 4). There was no significant difference between groups for PCS-12, MCS-12, GRCS and FABQ-W scores at any follow-up time points. However, there was a non-significant trend for more favourable GRCS scores in the PE plus MCE group compared with the PE alone group at the 20-week follow-up (Table 4).

### Sensitivity analysis

Improvement in pain intensity and disability level across groups was not significantly affected by age, gender, BMI, low back pain duration, or educational level. Additionally, none of these variables were more than weakly correlated (*r* range = 0.27–0.29) with any of the primary outcomes, hence not included as covariates in the analyses.

Table 5 shows a crosstabulation for the chi-square analyses of participants reporting ≥ 30% MCID on the NPRS and ODI across time. Participants in the PE plus MCE group were significantly more likely to report ≥ 30% reduction in NPRS scores compared with those in either therapy alone groups at the 8-week (*P* = 0.04) and 20-week (*P* = 0.03) follow-ups. However, participants in the MCE alone group were significantly more likely to report ≥ 30% reduction in ODI scores than those in the PE plus MCE group and PE alone group at the 8-week (*P* = 0.01) and 20-week (*P* = 0.03) follow-ups.

**Table 5:** Contingency table for reporting ≥ 30% minimal clinically important difference in pain intensity and disability level at 8 and 20 weeks

Variables	Time	30% MCID	PE plus MCE *n* (%)	PE *n* (%)	MCE *n* (%)	χ^2^	*P value*
**NPRS (0–10)**	8 weeks	Benefit	30 (75.0)	20 (50.0)	21 (59.2)	6.27	0.04*
		No benefit	10 (25.0)	20 (50.0)	19 (40.8)		
	20 weeks	Benefit	31 (77.5)	20 (50.0)	26 (65.0)	6.59	0.03*
		No benefit	9 (22.5)	20 (50.0)	14 (35.0)		
**ODI (0–100)**	8 weeks	Benefit	23 (57.5)	17 (42.5)	30 (75.0)	8.70	0.01*
		No benefit	17 (42.5)	23 (57.5)	10 (25.0)		
	20 weeks	Benefit	26 (65.0)	19 (47.5)	30 (75.0)	6.61	0.03*
		No benefit	14 (35.0)	21 (52.5)	10 (25.0)		

Benefit indicates participants reaching or exceeding 30% reduction in NPRS or ODI scores from baseline

No benefit indicates participants not reaching 30% reduction in NPRS or ODI scores from baseline

*NPRS* Numeric pain rating scale, *ODI* Oswestry disability index, *PE* Patient education, *MCE* Motor control exercise, *MCID* Minimal clinically important difference

The results of the per-protocol (see Supplementary Table 1, Additional file 3) and intention-to-treat analyses (Table 4) were nearly comparable in terms of significant between-group difference in primary and secondary outcomes. However, adherent participants with exception of NPRS scores had greater improvements in all the outcomes compared with the full participants. Additionally, there was a significant between-group difference favouring adherent participants in the PE plus MCE group compared with those in the PE alone group for GRCS scores at the 20-week follow-up and compared with those in the MCE alone group for BBQ scores at the 8-week follow-up.

## Discussion

### Summary of findings

To our knowledge, this is the first powered RCT to evaluate the effectiveness of adding PE to MCE compared with either therapy alone among community-dwelling adults with CLBP in a rural Nigerian or African context. The population of this study can be said to be a true representative of rural Nigerian dwellers as the majority of them were non-literates in formal education, practicing peasant or subsistence farming and suffering from long term low back pain. Additionally, fewer of them had hypertension and diabetes comorbidities, which are considered the most prevalent non-communicable diseases (31% and 5.7% for hypertension and diabetes respectively) in Nigeria [79, 80]. Our results showed in the short-term, greater improvements in pain intensity, disability level, fear-avoidance beliefs about physical activity and pain medication use in favour of PE plus MCE compared with PE alone; a greater improvement in disability in favour of MCE alone compared with PE alone; and greater improvements in pain catastrophising and back consequences belief in favour of PE alone compared with PE plus MCE and MCE alone.

### Pain intensity and disability

Given that CLBP is widely recognised as a complex biopsychosocial disorder [3], with pain and disability being the most important symptoms, research into context-specific, multidimensional treatment targeting both biological and psychosocial aspects of this disorder has been advocated [3, 81, 82]. Our study illustrates that combining PE with MCE significantly ameliorates pain and disability compared to using PE alone. However, since using MCE alone confers a better effect for disability compared to using PE alone, the use of exercise alone seems to be more cost-efficient in tackling CLBP disability. Importantly, the magnitude of the group differences in pain intensity (1.1–1.2 points) and disability level (5.0–6.5% points) were equal to or slightly above the MCID threshold values (1.0 points for pain and 5.0% points for disability) that were used to power the study. We opted to use smaller MCID values since smaller values may be appropriate for a simple, cheap and safe intervention, whereas larger values may be more appropriate for an expensive and risky intervention like surgery [56]. Moreover, our responder analyses illustrated that a greater number of the participants (> 57%) in the combined group and MCE alone group attained a clinically meaningful improvement. The superiority of PE plus MCE and MCE alone over PE alone is not quite so surprising in view of the fact that PE alone may not be sufficient to address the complexity of CLBP [81]. It is worth noting that the improvement in pain intensity and disability level achieved by the participants in the PE alone group may have also been contributed by the stretching and aerobic exercises used as adjunct therapies by all the groups. While trials comparing PE plus MCE with PE alone are limited in the literature, our results are in agreement with those reported by previous trials conducted in urban European [83, 84] and Asian [85, 86] settings using pain neuroscience education (PNE) – another form of biopsychosocial education, combined with different forms of exercise therapy. For example, Malfliet et al. [84] showed that a 12-week combined PNE and MCE programme resulted in a greater alleviation of pain and disability compared with evidence-based physiotherapy in patients with chronic spinal pain. Rabie et al. [85] showed a greater improvement in similar outcomes with an 8-week individualised PNE plus MCE programme compared with group-based general exercise. Greater effects were also demonstrated in favour of a similar combination approach compared with core strengthening exercise among women with CLBP [86]. However, our results appear not to be in concurrence with those reported by Pardo et al. [87] demonstrating superior effect of a 12-week PNE plus multimodal exercise programme (i.e. MCE, stretching, and aerobics) compared with the same multimodal exercise programme. Additionally, and surprisingly, a pilot study by Ryan et al. [88] showed that PNE alone was superior for disability compared with PNE plus exercise therapy class at the end of 6-week intervention but improvement leveled off at 12-week follow-up. It should be noted that variation in results across studies might be due to variations in the exercise and education programmes employed besides the differences in the intervention dosage and types of outcome measures used (e.g. Roland-Morris disability questionnaire in place of the ODI).

### Quality of life

Improving the QoL of individuals with CLBP disorder is pivotal in rehabilitation as pain and disability often interfere with daily activities of living and so reducing QoL [89]. Combined PNE and MCE has been shown to be more effective in improving physical and mental health compared with current best-evidence physiotherapy in patients with chronic spinal pain [84]. Moreover, MCE alone resulted in better physical health compared with daily walks in patients with CLBP [90]. However, the comparative effectiveness of PE plus MCE vs PE alone vs MCE alone on physical and mental health outcomes has not been previously reported. In the present study, the lack of difference observed between the groups in both physical and mental health scores is somewhat surprising given that PE alone, in particular, is not expected to sufficiently improve QoL. However, the addition of stretching and aerobic exercises could have accounted for the improvement in the PE alone group though mental health was not maintained at the 20-week follow-up.

### Global perceived recovery

Improvement in global perceived effect in all the groups with no significant difference observed between the groups at any follow-up time points further substantiates the usefulness of each of the study interventions. However, participants in the combined group had slightly better GRCS scores relative to those in the PE alone group at the 20-week follow-up, which could have possibly been due to the greater improvement in pain and disability achieved by the combined group. This was confirmed by the per-protocol analysis. The GRCS has not been commonly used as an outcome measure in trials examining the effectiveness of cognitive-based PE plus MCE [81, 84, 86, 88]. However, Pardo et al. [87] reported a greater perceived improvement, as measured by the 7-point GRCS, in favour of PNE plus multimodal exercise programme compared with the same multimodal exercise programme.

### Fear-avoidance beliefs

Fear-avoidance beliefs have been reported to be among the important predictors of disability in rural Nigeria [4], and decreasing these negative beliefs has been associated with decreased pain and disability [91, 92]. The improvement observed in both FABQ-PA and FABQ-W scores in all the groups in the present study is, therefore, crucial and such improvement could be said to have been reflected in the pain and disability outcomes. The greater improvement observed in favour of the combined group compared with the PE or MCE alone group for FABQ-PA scores though not maintained at the 20-week follow-up, might have been due to the interaction between PE and MCE. The PE is believed to have altered the participants’ attitudes and beliefs about pain and movement, thus, helping them progressively return to those activities/movements considered as being fearful and doubtful to execute. This is further augmented by performing MCE. The result that the groups were not significantly different in FABQ-W scores may be partially explained by the fact that only 7.5% of the studied population were workers, hence, the work subscale may not be too relevant to the population. Moreover, it is likely that the participants could not discriminate between physical activity and work since the majority of them were manual labourers and their job activities involved physical movements and activity. In line with our study, a previous trial [85] found both PNE plus MCE and trunk strengthening exercise to be effective at improving FABQ-W scores in patients with CLBP.

### Pain catastrophising

Catastrophising has been also reported as one of the significant predictors of disability in rural Nigeria [4]. While all the intervention strategies improved pain catastrophising, interestingly, the use of PE alone proved to be more effective compared to combining PE and MCE. This is rather surprising in view of the fact that combining education with exercise may seem to confer more benefits than using education alone. Nonetheless, It could not be excluded that the interaction of PE with MCE might have distracted the messages provided in the PE sessions and augmented the participants’ perception of being patients, with a biomedical (i.e. structural) problem as they had more exposure to MCE sessions (mean MCE sessions = 9.1 vs mean PE sessions = 4.7). Thus, this may explain, at least partly, the greater improvement in the PE alone group. It is believed that our PE programme may have impacted positively on pain catastrophising through pain reconceptualisation and modification of maladaptive pain cognitions. Consistent with our results, Moseley et al. [34] reported a significant reduction in PCS scores (5.0 points) in favour of intensive PNE compared to a biomedical-based PE among patients with CLBP. However, our study resulted in a larger effect (12.0 points).

### Back pain consequences belief

Modifying negative beliefs about low back pain is fundamental; especially among low-literate individuals since low education levels may be considerably associated with these beliefs. A prior trial [93] showed that a PNE programme could have a positive impact on patients’ beliefs outcomes including back pain consequences belief. Moreover, a previous systematic review [36] and state of art review [37] suggest PE based on the biopsychosocial framework to shift patients’ beliefs on low back pain. In our study, participants receiving PE alone had better improvement in BBQ scores compared with those receiving PE plus MCE or MCE alone. The PE programme in the present study included information aiming to modify false beliefs about low back pain, thus participants receiving PE are expected to have better BBQ scores compared to those receiving MCE only. However, the superiority of the PE alone over the PE plus MCE could possibly have been due to the addition of MCE which may have diluted some of the messages put forward in the PE programme as earlier thought.

### Pain medication use

The use of pain tablets decreased in all groups at the end of the intervention, but superior improvement was observed for the PE plus MCE compared with the PE alone. This could have been related to the positive reinforcement effects as a result of combining PE with MCE leading to better pain relief as evidenced by the significant reduction in the NPRS scores. Pain medication dependency has been reported to be a salient maladaptive coping strategy among adult rural dwellers in Nigeria [21]. Our PE programme was targeted at reducing such maladaptive coping strategies by promoting active coping strategies and self-management skills. This was further enhanced by performing structured MCE training. Reduction in the utilisation of pain medication, especially opioids is of great economical and public health importance given the cost, potential adverse effects and risk of addiction associated with long-term use [94].

### Strengths and limitations

As regards the strength of this study, the results are justified by some important methodological features known to minimise bias in clinical trials. These features include RCT design, power analysis, concealed allocation, blind outcome assessment and intention-to-treat analysis. Additionally, per-protocol and responder analyses were conducted.

The present study also had some limitations which should be considered when interpreting the results. First, our study had a substantial attrition rate, particularly during the 20-week follow-up (> 20%). However, the results of the intention-to-treat analysis were nearly comparable to those of the per-protocol analysis. Second, outcomes were only evaluated in the shorter-term, and longer-term effect is unknown. Third, we did not include a control group which would have allowed estimation of truly significant effects and minimise the assumption that improvement may be attributable to the natural course of low back pain. Fourth, we could not preclude potential contamination of the study interventions with other pain treatments even though the participants were informed to refrain from such treatments. Fifth, self-report questionnaires were used as outcome measures, which may be subject to recall bias from the participants. Lastly, it was difficult to compare the results of the current study with previous studies as the content of the interventions, especially the PE programme varied markedly.

### Implications for clinical practice and future research

Although adding PE to MCE in the current trial reflects current clinical guideline recommendations [28, 29] and importantly, appears to alleviate pain and disability associated with CLBP better than using PE alone, the use of MCE alone may be cost-efficient since it provides a comparable effect with the combination strategy on disability. Additionally, PE alone seems to be more cost-efficient to alter maladaptive pain-related beliefs and cognitions, though some patients requiring more support may need additional treatment with MCE. Given that combined PE and MCE also provide superior effects on other important outcomes for example fear-avoidance beliefs about physical activity and pain medication use, besides being safe and inexpensive, such intervention strategy may influence or guide rehabilitation professionals on the choice of effective intervention to tackle CLBP and promote self-management in rural or low-resource settings. The present study may also pave the way for future research in other rural contexts, especially in Africa.

## Conclusions

Among rural community-dwelling adults with CLBP, PE plus MCE led to greater short-term improvements in pain and disability compared with PE alone, although all intervention strategies were associated with improvements in these outcomes. This trial provides additional support for combining PE with MCE, as recommended in current clinical guidelines, to promote self-management and reduce burden of CLBP in low-resource rural communities. Further research, which is underway, is needed to determine long-term effectiveness of these interventions.

## Supplementary Information

**Additional file 1: Supplementary Figure 1.** Stretching exercise programme.

**Additional file 2: Supplementary Figure 2.** Motor control exercise programme.

**Additional file 3: Supplementary Table 1.** Intervention effectiveness on primary and secondary outcomes analysed based on per-protocol principle.

## Data Availability

Data will be made available on request from the corresponding author.

## Abbreviations

ADIM: Abdominal drawing-in manoeuvre
ANOVA: Analysis of variance
BBQ: Back beliefs questionnaire
BMI: Body mass index
CLBP: Chronic low back pain
FABQ: Fear-avoidance beliefs questionnaire
FABQ-PA: Fear-avoidance beliefs questionnaire – (physical activity subscale)
FABQ-W: Fear-avoidance beliefs questionnaire – (work subscale)
GRCS: Global rating of change scale
LMM: Linear mixed-effects model
MCE: Motor control exercise
MCID: Minimal clinically important difference
MCS-12: Mental component scores
NPRS: Numerical pain rating scale
ODI: Oswestry disability index
PCS: Pain catastrophising scale
PCS-12: Physical component scores
PE: Patient education
PNE: Pain neuroscience education
QoL: Quality of life
RCT: Randomised clinical trial
SD: Standard deviation
SF-12: 12-item short form health survey
