# Supplementary figures and images for: Effectiveness of patient education plus motor control exercise versus patient education alone versus motor control exercise alone for rural community-dwelling adults with chronic low back pain: a randomised clinical trial
Source: BMC Musculoskelet Disord. 2023 Feb 23;24:142. doi: 10.1186/s12891-022-06108-9 (PMC9948461; doi:10.1186/s12891-022-06108-9)

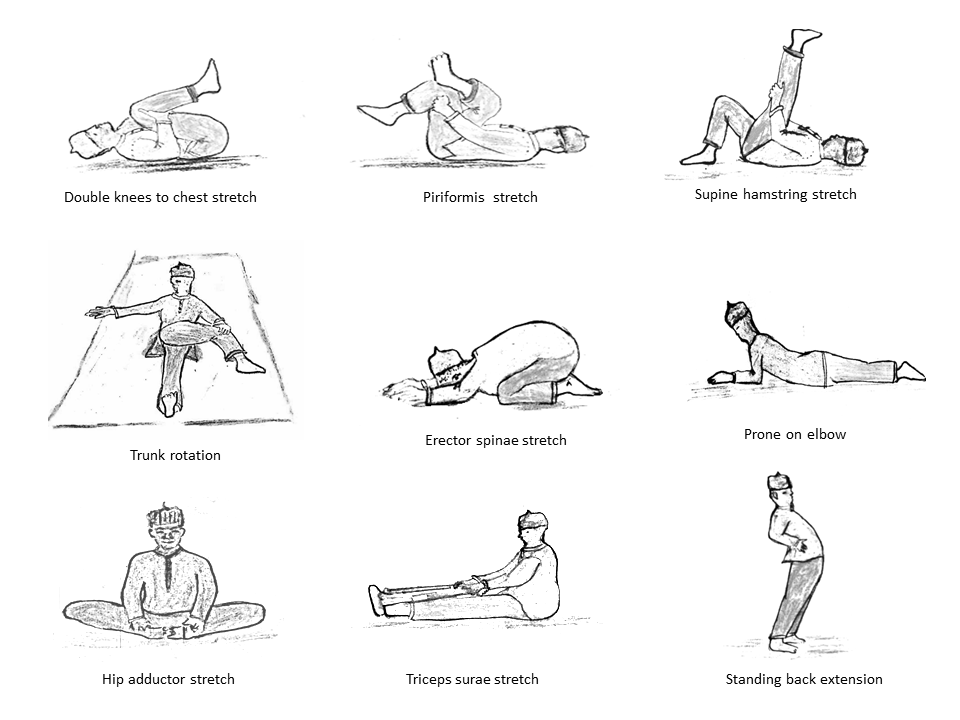

Supplement: Supplementary file 1 — Additional file 1: Supplementary Figure 1. Stretching exercise programme. [file 12891_2022_6108_MOESM1_ESM.tif]
