# Supplementary material for: Effectiveness of patient education plus motor control exercise versus patient education alone versus motor control exercise alone for rural community-dwelling adults with chronic low back pain: a randomised clinical trial
Source: BMC Musculoskelet Disord. 2023 Feb 23;24:142. doi: 10.1186/s12891-022-06108-9 (PMC9948461; doi:10.1186/s12891-022-06108-9)
Supplement: Supplementary file 2 — Additional file 2: Supplementary Figure 2. Motor control exercise programme. [file 12891_2022_6108_MOESM2_ESM.pdf]

STAGE ONE

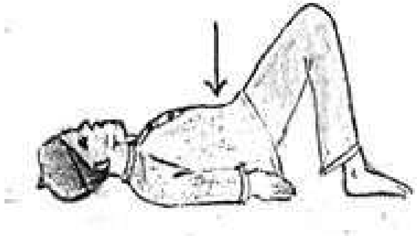

ADIM in lying

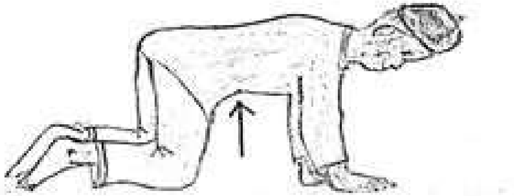

ADIM in quadruped

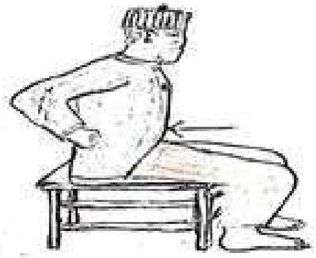

ADIM in sitting

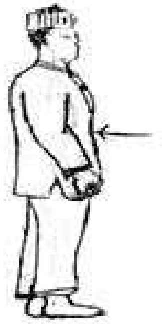

ADIM in standing

STAGE TWO

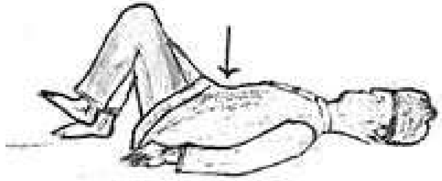

Supine ADIM with leg lift

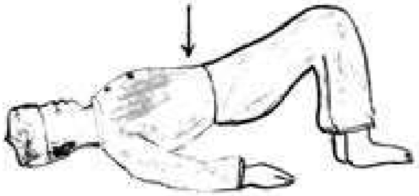

Supine ADIM with two-leg bridge

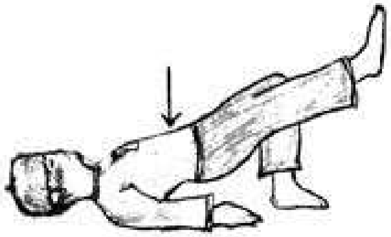

Supine ADIM with one-leg bridge

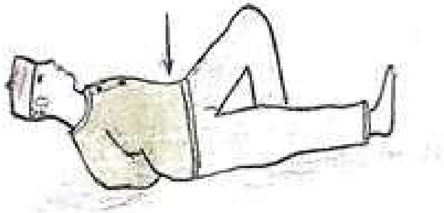

Supine ADIM with curl-up and elbows on the table

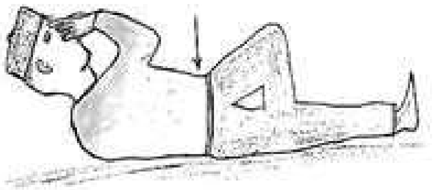

Supine ADIM with curl-up and hands over the forehead

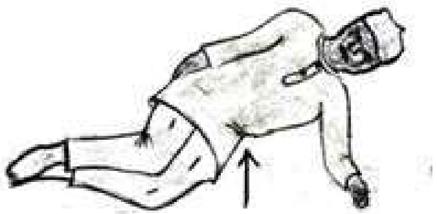

ADIM in horizontal side support with knees bent

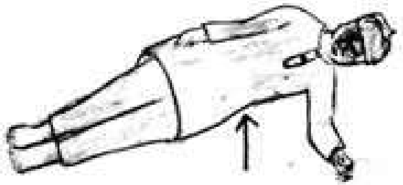

ADIM in horizontal side support with knees staright

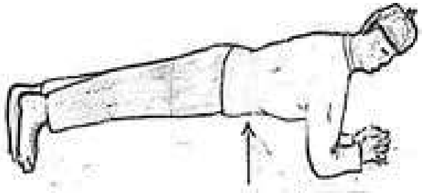

Side-lying horizontal side support with ADIM

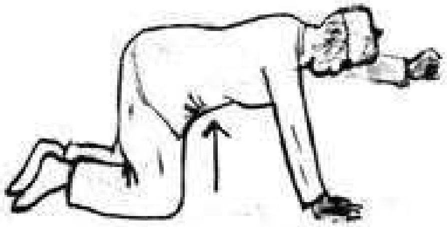

ADIM in quadruped with arm raise

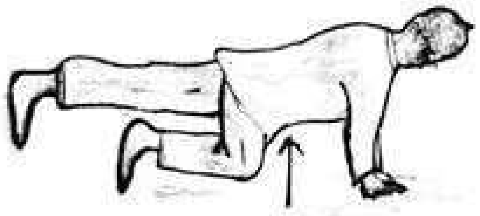

ADIM in quadruped with leg raise

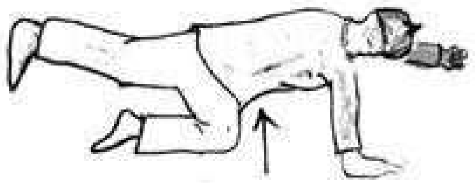

ADIM in quadruped with alternate arm and leg raise

STAGE THREE

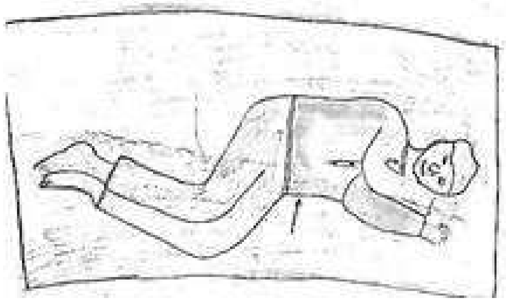

Rolling from side to side with ADIM

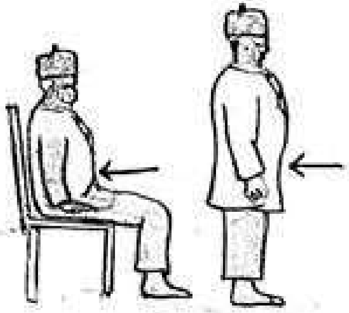

Sit to stand with ADIM

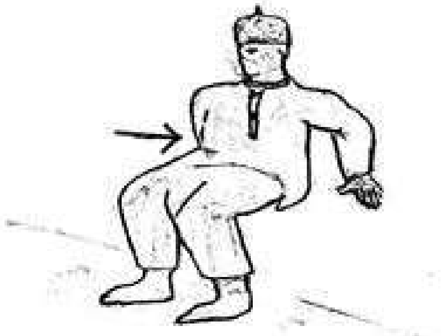

Wall squatting with ADIM

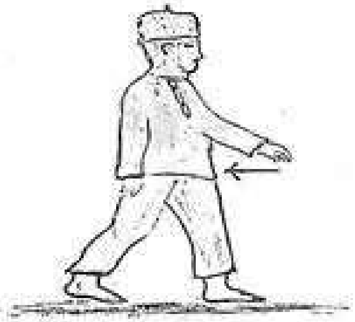

Walking with ADIM
