# Supplementary material for: Effectiveness of patient education plus motor control exercise versus patient education alone versus motor control exercise alone for rural community-dwelling adults with chronic low back pain: a randomised clinical trial
Source: BMC Musculoskelet Disord. 2023 Feb 23;24:142. doi: 10.1186/s12891-022-06108-9 (PMC9948461; doi:10.1186/s12891-022-06108-9)
Supplement: Supplementary file 3 — Additional file 3: Supplementary Table 1. Intervention effectiveness on primary and secondary outcomes analysed based on per-protocol principle. [file 12891_2022_6108_MOESM3_ESM.docx]

**Additional file 3: Supplementary Table 1 Intervention effectiveness on primary and secondary outcome analysed based on per-protocol analysis**

| **Outcome** | **Treatment group**  Mean (SD) | | | **Adjusted between-group difference**  Mean (95% CI) | | |
| --- | --- | --- | --- | --- | --- | --- |
|  | PE+MCE | PE | MCE | PE+MCE vs PE | PE+MCE vs MCE | PE vs MCE |
| **Primary outcomes** |  |  |  |  |  |  |
| **NPRS (0–10)** |  |  |  |  |  |  |
| Baseline | 6.41 (1.32) | 6.41 (1.79) | 2.94 (1.43) | – | – | – |
| 8 weeks | 3.00 (1.41) | 4.05 (1.55) | 3.06 (1.86) | –1.04 (–2.30 to –0.21) | –0.05 (–1.37 to 1.26) | 0.99 (–0.24 to 2.27) |
| 20 weeks | 2.94 (1.43) | 4.09 (1.74) | 3.33 (1.18) | –1.15 (–2.29 to –0.00)* | –0.39 (–1.58 to –0.80) | 0.75 (–0.36 to 1.88) |
| **ODI (0–100)** |  |  |  |  |  |  |
| Baseline | 22.0 (10.2) | 25.9 (13.9) | 24.7 (12.5) | – | – | – |
| 8 weeks | 12.1 (6.36) | 17.8 (9.28) | 13.0 (7.32) | –5.76 (–12.9 to 1.42) | –0.90 (–8.43 to 6.61) | 4.85 (–2.21 to 11.9) |
| 20 weeks | 9.72 (4.92) | 17.9 (9.17) | 10.5 (5.22) | –8.17 (–15.3 to –0.98)* | –0.82 (–8.35 to 6.70) | –7.34 (0.27 to 14.4)* |
| **Secondary outcomes** |  |  |  |  |  |  |
| **PCS-12 (0–100)** |  |  |  |  |  |  |
| Baseline | 36.4 (7.90) | 35.9 (7.49) | 36.9 (7.47) | – | – | – |
| 8 weeks | 43.5 (7.83) | 43.0 (7.45) | 44.2 (4.96) | 0.41 (–3.58 to 4.40) | –0.74 (–4.92 to 3.44) | –1.15 (–5.08 to 2.77) |
| 20 weeks | 47.9 (4.02) | 45.5 (4.85) | 45.9 (3.82) | 1.33 (–2.66 to 5.32) | –1.93 (–2.24 to 6.11) | 0.60 (–3.32 to 4.53) |
| **MCS-12 (0–100)** |  |  |  |  |  |  |
| Baseline | 40.5 (7.57) | 41.5 (11.5) | 40.2 (9.95) | – | – | – |
| 8 weeks | 45.9 (8.28) | 49.9 (9.55) | 47.5 (4.43) | –4.01 (–9.19 to 1.16) | –1.67 (–7.10 to 3.74) | 2.33 (–2.75 to 7.43) |
| 20 weeks | 47.9 (4.02) | 46.5 (4.85) | 45.9 (3.82) | 0.15 (–5.33 to 5.02) | 0.09 (–5.33 to 5.51) | 0.24 (–4.84 to 5.34) |
| **GRCS (–5 to +5)** |  |  |  |  |  |  |
| Baseline | –1.24 (1.67) | –1.41 (1.99) | –1.11 (1.53) | – | – | – |
| 8 weeks | 3.00 (1.11) | 2.77 (1.80) | 2.22 (1.73) | 0.72 (–0.26 to –1.71) | 0.77 (–0.25 to 1.81) | 0.05 (–0.92 to 1.02) |
| 20 weeks | 3.00 (1.27) | 1.59 (1.59) | 2.00 (1.18) | 1.40 (0.42 to 2.39)** | 1.00 (–0.03 to 2.03) | –0.40 (–1.38 to 0.56) |
| **FABQ-PA (0–24)** |  |  |  |  |  |  |
| Baseline | 14.0 (7.42) | 12.9 (7.27) | 13.4 (6.68) | – | – | – |
| 8 weeks | 5.94 (5.59) | 8.95 (4.98) | 10.2 (4.15) | –3.01 (–6.49 to 0.47) | –4.33 (–7.98 to –0.68) | –1.32 (–4.75 to 2.10) |
| 20 weeks | 4.73 (3.93) | 6.68 (4.15) | 8.17 (4.21) | –2.36 (–6.07 to 1.28) | –3.55 (–7.30 to 0.19) | –1.15 (–4.68 to 2.37) |
| **FABQ-W (0–42)** |  |  |  |  |  |  |
| Baseline | 25.4 (9.50) | 25.1 (9.24) | 24.5 (9.95) | – | – | – |
| 8 weeks | 15.9 (8.70) | 14.4 (8.88) | 17.0 (8.19) | 1.48 (–3.61 to 6.58) | 1.05 (–6.40 to 4.28) | –2.54 (–7.56 to 2.47) |
| 20 weeks | 12.6 (7.14) | 8.53 (4.11) | 10.8 (5.82) | 4.73 (–0.66 to 10.1) | 2.38 (–3.10 to 7.88) | –2.34 (–7.51 to 2.82) |
| **PCS (0–52)** |  |  |  |  |  |  |
| Baseline | 32.3 (6.17) | 29.4 (5.73) | 33.4 (8.66) | – | – | – |
| 8 weeks | 20.0 (7.25) | 15.5 (6.50) | 20.0 (6.50) | 4.46 (0.47 to 8.46)* | 0.00 (–4.17 to 4.18) | –4.46 (–8.39 to –0.53)* |
| 20 weeks | 18.1 (6.13) | 12.0 (5.97) | 15.9 (3.88) | 6.04 (1.78 to 10.3)** | 2.35 (–2.04 to 6.74) | –3.69 (–7.77 to 0.38) |
| **BBQ (9–45)** |  |  |  |  |  |  |
| Baseline | 17.3 (5.52) | 17.5 (5.11) | 16.1 (3.68) | – | – | – |
| 8 weeks | 27.1 (5.51) | 29.9 (5.76) | 22.8 (4.80) | 2.83 (–5.90 to 0.22) | 4.28 (1.07 to 7.49)** | 7.12 (4.10 to 10.1)*** |
| 20 weeks | 29.8 (4.62) | 33.1 (4.05) | 27.6 (4.79) | –3.34 (–6.68 to –0.10)* | 2.19 (–1.18 to 5.57) | 5.54 (2.42 to 8.65)** |
| **Pain medication use** |  |  |  |  |  |  |
| Baseline | 8.52 (8.68) | 8.77 (5.98) | 12.4 (9.23) | – | – | – |
| 8 weeks | 2.70 (3.93) | 10.4 (6.70) | 8.66 (7.73) | –7.74 (–12.2 to –3.25)** | –5.96 (–10.6 to –1.25)* | 1.78 (–2.63 to 6.20) |

*CI* Confidence interval, *PE* Patient education, *MCE* Motor control exercise, *NPRS* Numerical pain rating scale, *ODI* Oswestry disability index, *PCS-12* Physical component summary-12, *MCS-12* Mental health component summary-12, *GRCS* Global rating of change scale, *FABQ-PA* Fear-avoidance beliefs questionnaire – (physical activity), *FABQ-W*  Fear-avoidance beliefs questionnaire – (work), *PCS* Pain catastrophising scale, *BBQ* Back beliefs questionnaire, *NA* Not applicable

Lower scores in NPRS, ODI, FABQ-PA, FABQ-W, and PCS indicate better improvement

Higher scores in PCS-12, MCS-12, GRCS, and BBQ indicate better improvement

Participants in PE plus MCE group = 17, PE group = 22, and MCE group = 18

* comparison is significant at the 0.05 level,

** comparison is significant at the 0.01 level

*** comparison is significant at the 0.001 level
